# Dietary Green Pea Protects against DSS-Induced Colitis in Mice Challenged with High-Fat Diet

**DOI:** 10.3390/nu9050509

**Published:** 2017-05-18

**Authors:** Shima Bibi, Luís Fernando de Sousa Moraes, Noelle Lebow, Mei-Jun Zhu

**Affiliations:** School of Food Science, Washington State University, Pullman, WA 99164, USA; Shima.bibi@wsu.edu (S.B.); nandomoraesufv@yahoo.com.br (L.F.d.S.M.); noelle.lebow@wsu.edu (N.L.)

**Keywords:** high-fat diet, colitis, green pea, inflammation, mucin 2, endoplasmic reticulum stress

## Abstract

Obesity is a risk factor for developing inflammatory bowel disease. Pea is unique with its high content of dietary fiber, polyphenolics, and glycoproteins, all of which are known to be health beneficial. We aimed to investigate the impact of green pea (GP) supplementation on the susceptibility of high-fat diet (HFD)-fed mice to dextran sulfate sodium (DSS)-induced colitis. Six-week-old C57BL/6J female mice were fed a 45% HFD or HFD supplemented with 10% GP. After 7-week dietary supplementation, colitis was induced by adding 2.5% DSS in drinking water for 7 days followed by a 7-day recovery period. GP supplementation ameliorated the disease activity index score in HFD-fed mice during the recovery stage, and reduced neutrophil infiltration, mRNA expression of monocyte chemoattractant protein-1 (MCP-1) and inflammatory markers interleukin (IL)-6, cyclooxygenase-2 (COX-2), IL-17, interferon-γ (IFN-γ), and inducible nitric oxide synthase (iNOS) in HFD-fed mice. Further, GP supplementation increased mucin 2 content and mRNA expression of goblet cell differentiation markers including Trefoil factor 3 (Tff3), Krüppel-like factor 4 (Klf4), and SAM pointed domain ETS factor 1 (Spdef1) in HFD-fed mice. In addition, GP ameliorated endoplasmic reticulum (ER) stress as indicated by the reduced expression of Activating transcription factor-6 (ATF-6) protein and its target genes chaperone protein glucose-regulated protein 78 (Grp78), the CCAAT-enhancer-binding protein homologous protein (CHOP), the ER degradation-enhancing α-mannosidase-like 1 protein (Edem1), and the X-box binding protein 1 (Xbp1) in HFD-fed mice. In conclusion, GP supplementation ameliorated the severity of DSS-induced colitis in HFD-fed mice, which was associated with the suppression of inflammation, mucin depletion, and ER stress in the colon.

## 1. Introduction

According to the latest NHANES survey (2009–2010), 31.9% of non-pregnant women 20–39 years of age are obese, and another one-third are overweight [1]. In parallel with the increased obesity prevalence, the incidence of inflammatory bowel disease (IBD), consisting of Crohn’s disease (CD) and ulcerative colitis (UC), is on the rise. IBD is a chronic relapsing disorder of the gut with a complicated etiology. Increasing evidence indicates that Western dietary and life-style habits contribute to the increased prevalence of IBD by inducing intestinal inflammation [2]. 

The Western diet is high in fat and low in fiber, which aggravates dextran sodium sulfate (DSS)-induced colitis [3], and is further exacerbated by the intake of red meat [4]. Long-term high intake of trans-unsaturated fats is associated with an increased risk of UC in women in the USA [5]. Recently, we found that maternal HFD consumption during gestation and lactation predisposed female offspring to a higher susceptibility to DSS-induced colitis through increased inflammatory responses [6]. HFD consumption also induces oxidative and endoplasmic reticulum (ER) stress [7], leading to mucin 2 protein misfolding in cultured colon cells [7]. Mucin 2 depletion and misfolding correlates with colitis in mice [8]. 

On the contrary to the HFD, high vegetable and fiber intake is associated with a decreased risk of IBD [9]. Epidemiologically, legume intake was protective against colorectal cancer in a case control study [10], and significantly reduced the risk of colorectal adenoma in a meta-analysis of three cohort studies and eleven case control studies [11]. Legumes and pulses, including peas, are rich in fiber and other phytonutrients that boost beneficial intestinal microbiota [12], producing short chain fatty acids (SCFA) and promoting epithelial barrier integrity [13]. Further, dietary soybean Bowman–Birk inhibitor concentrate [14], white and dark kidney beans [15], and cranberry bean supplements [16] suppressed colonic inflammation and reduced the severity of DSS-induced colitis in mice. Consistently, pea seed albumin extract ameliorated DSS-induced colitis in mice by reducing the expression of inflammatory markers in colonic tissues [17]. These results suggest that beans in general might have protective effects against colitis. The objective of the current study was to investigate the preventive effect of dietary green pea (GP) supplementation on DSS-induced colitis in HFD-fed female mice and further examine its underlying mechanism. 

## 2. Materials and Methods

### 2.1. Green Pea (GP)

GP was purchased from Moscow Food Co-op (Moscow, ID, USA) and powdered in the cyclone mill (Model 3010-060, UDY Corp., Fort Collins, CO, USA). The powdered GP was shipped to the Research Diets, Inc. (New Brunswick, NJ, USA) for customized diet formulation. 

### 2.2. Experimental Design and Animal Diets

Six-week-old C57BL/6J female mice (originally purchased from Jackson Laboratory, Bar Harbor, ME, USA, and inbred in our facility) were randomly divided into two groups. One group of mice (*n* = 7) was fed with the HFD (45% energy from fat, D12451, Research Diets Inc., New Brunswick, NJ, USA) (Table S1), and the other group of mice (*n* = 7) was fed HFD supplemented with GP (10% of dry feed weight) (HFDGP, D15080605, Research Diets Inc., New Brunswick, NJ, USA) (Table S1) for a total duration of 9 weeks. The dose of GP (10%) supplement was 100 g/kg of the diet. The average daily consumption by mice was 2.40 g/mouse. This equals to 240 mg GP per day for an adult mouse of 20 g (i.e., 12 g GP/day/kg body mass), which converts to 58.38 g of GP daily consumption for a 60 kg human per the published formula [18]. Colitis was induced using colitis grade DSS (Molecular Weight = 36,000–50,000) (MP Biomedicals, Santa Ana, CA, USA) after 7 weeks of dietary supplementation. Both groups were given 2.5% DSS (*w*/*v*) in drinking water for 7 days followed by a 7-day recovery period providing normal drinking water (Figure S1). We used only virgin females in the study to avoid a confounding sex effect and to minimize potential differences in female hormone cycling. Mice were monitored daily throughout the DSS treatment and recovery period for disease symptoms. All mice were housed in a temperature-controlled room with a 12 h light and 12 h dark cycle and had free access to diet and drinking water. No differences were observed in the average amount of water and feed consumption (Figure S2A) between treatment groups. All animal procedures were approved (BAF # 04316-010) by the Washington State University Animal Care and Use Committee.

### 2.3. Assessment of Colitis Symptoms and Disease Activity Index

Mice were monitored daily for body weight loss compared to initial weight (scored as 0–4), fecal consistency (scored as 0–4), and blood in the stool (scored as 0–4) throughout the DSS treatment and recovery period. The disease activity index (DAI) score was assessed as the combined score of the above three criteria [19]. 

### 2.4. Colonic Tissue Collection and Processing

Mice were anesthetized with CO_2_ inhalation and followed by cervical dislocation. The entire colon was dissected, and a 5 mm segment of the distal colon at a constant location was fixed in freshly prepared 4% (*w*/*v*) paraformaldehyde (pH 7.0), processed, and embedded in paraffin. The remaining colonic tissue, containing both inflamed and non-inflamed areas, was rinsed in PBS, frozen in liquid nitrogen, and stored at −80 °C for later biochemical analysis.

### 2.5. Neutrophil Assessment

Paraffin embedded tissues were cut into 5 µm thick sections, deparaffinized, and hydrated, followed by antigen retrieval, goat serum blocking, and overnight incubation with anti-Ly-6B.2 antibody (Bio-Rad Laboratories Inc., Hercules, CA, USA). After incubation with the secondary antibody, signals were visualized using the Vectastain ABC and DAB peroxidase (HRP) substrate kits (Vector Laboratories Inc., Burlingame, CA, USA) and haematoxylin counterstaining. Images were taken using the Lecia DM2000 LED light microscope (Chicago, IL, USA). Neutrophil infiltration scores were assessed blindly by two researchers using the criteria described previously [20]. Briefly, the scores for depth of neutrophil infiltration (scored as 0–3) and staining intensity (scored as 0–4), which was the percent area positive as extent (0, none; 1, <25%; 2, 25–50%; 3, 50–75%; 4, >75%), were recorded individually. The summation of both scores resulted in a total quantified score ranging from 0 to a maximum of 7 per distal colonic section. Nine sections per animal at constant interval were used for microscopic examination and score assessment.

### 2.6. Immunoblotting Analysis

Immunoblotting analyses were performed as previously described [21]. Band density was quantified using the Odyssey Infrared Imaging System and Image Studio™ Lite software (Li-Cor Biosciences, Lincoln, NE, USA), and normalized to the β-actin content. Antibodies against activating transcription factor-6 (ATF-6), mucin 2, and xanthine oxidase (XO) were from Santa Cruz Biotechnology Inc. (Dallas, TX, USA). Cyclooxygenase-2 (COX-2) and interleukin (IL)-6 primary antibodies were purchased from Cell Signaling Technology (Beverly, MA, USA). Anti-β-actin antibody was from Sigma (St. Louis, MO, USA). IRDye 680 goat anti-mouse and IRDye 800CW goat anti-rabbit secondary antibodies were purchased from Li-Cor Biosciences (Lincoln, NE, USA).

### 2.7. qRT-PCR Analysis

Total RNA was extracted from the powdered colonic tissue using Dynabeads^®^ mRNA DIRECT™ Purification Kit (Invitrogen, Carlsbad, CA, USA) following the protocol of the manufacturer. cDNA was synthesized with the iScript™ cDNA synthesis kit (Bio-Rad Laboratories Inc., Hercules, CA, USA). qRT-PCR was performed on a Bio-Rad CFX384 real-time thermocycler [22]. The 18S was used as the reference gene. Primer sequences are provided in Table S2.

### 2.8. Statistical Analysis

All data were analyzed as a complete randomized design using the General Linear Model of Statistical Analysis System (2000), expressed as mean ± standard error of mean (SEM). Student’s *T*-test was used for calculating significance. A significant difference was considered as *p* ≤ 0.05.

## 3. Results

### 3.1. GP Supplementation Counteracts Symptoms of DSS-Induced Colitis in HFD-Fed Mice

**Figure 1:**
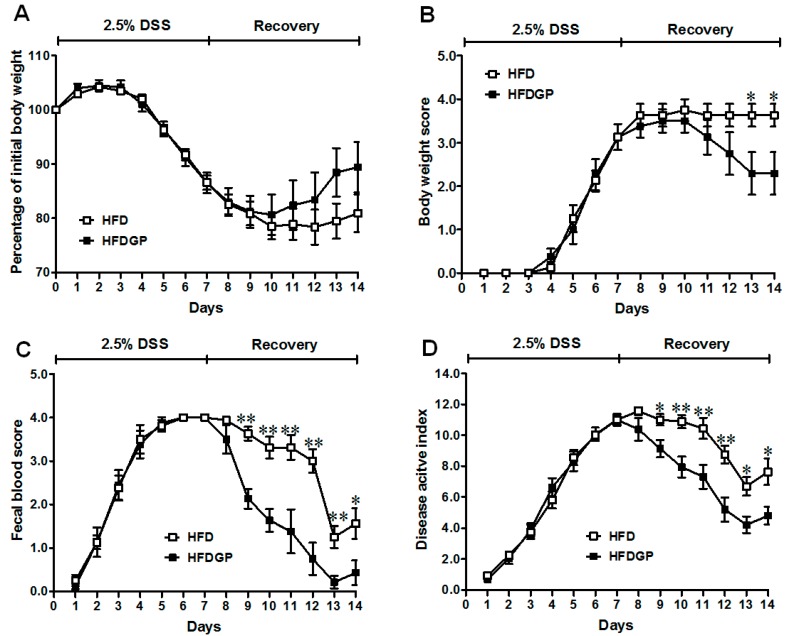
Symptoms of dextran sulfate sodium (DSS)-induced colitis in high-fat diet (HFD) (□) or HFD supplemented with green pea (HFDGP) (■) fed mice. (**A**) Body weight loss; (**B**) Body weight loss score; (**C**) Fecal blood score; (**D**) Disease activity index score during DSS treatment and recovery process; a higher score correlates with severer symptoms. Means ± SEM, *n* = 7, * *p* ≤ 0.05, ** *p* ≤ 0.01.

DSS-induction caused colitis symptoms in mice. The HFD-fed mice with and without GP supplementation showed similar symptomatic parameters during the DSS-treatment phase (Figure 1). However, during the recovery phase, the GP-supplemented HFD-fed group recovered faster than mice without GP supplementation. The body weight loss and body weight loss score remained lower in the GP-supplemented HFD-fed group throughout the recovery period (Figure 1A,B). Further, a significant reduction in the fecal blood and DAI score was found in GP-supplemented HFD-fed mice (Figure 1C,D). There was no difference in body weight between the two groups before DSS-induction (Figure S2B). 

### 3.2. GP Supplementation Reduces Neutrophil Recruitment and Monocyte Chemoattractant Protein-1 (MCP-1) Expression in HFD-Fed DSS-Colitis Mice

**Figure 2:**
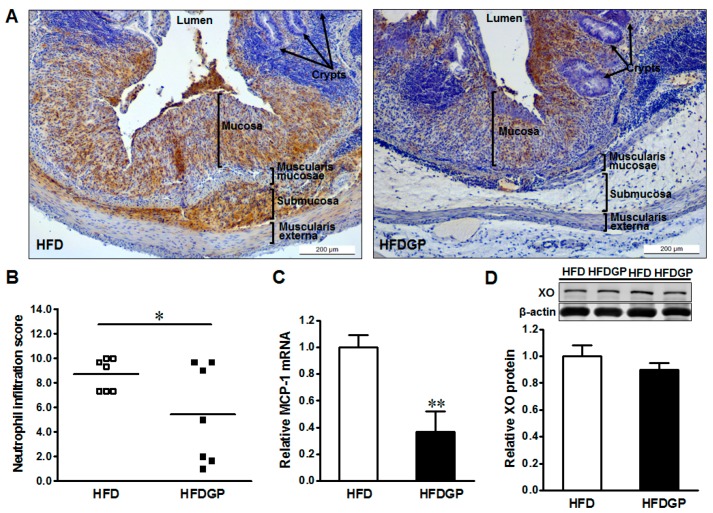
Immunohistochemical staining of neutrophils in distal colonic tissues of high-fat diet (HFD) (□) or high-fat diet supplemented with green pea (HFDGP) (■) fed DSS-colitis mice. (**A**) Representative images of neutrophil staining; (**B**) Neutrophil quantified score; (**C**) mRNA expression of MCP-1; (**D**) Representative immunoblotting bands and statistical data of xanthine oxidase (XO). Means ± SEM, *n* = 7, * *p* ≤ 0.05, ** *p* ≤ 0.01.

GP supplementation reduced the neutrophil recruitment, and resultant tissue damage in the colonic tissues of HFD-fed DSS-colitis mice (Figure 2A,B). In accordance, GP supplementation reduced the gene expression of MCP-1 (Figure 2C), which enhances the recruitment of neutrophils into the mesenteric tissues [23].

### 3.3. GP Supplementation Reduces Inflammation and Oxidative Stress in HFD-Fed DSS-Colitis Mice

**Figure 3:**
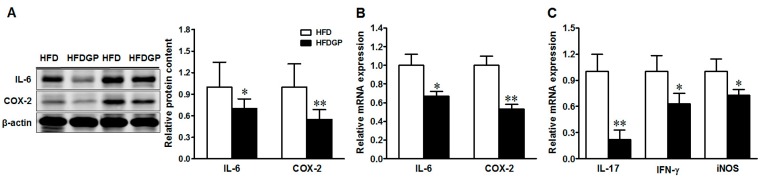
Inflammatory mediators in the colon of HFD or HFDGP fed DSS-colitis mice. (**A**) Representative immunoblotting bands and statistical data of IL-6 and COX-2; (**B**) mRNA expression of IL-6 and COX-2; (**C**) mRNA expression of IL-17, IFN-γ and iNOS. Means ± SEM, *n* = 7, * *p* ≤ 0.05, ** *p* ≤ 0.01.

In agreement with improved epithelial damage, GP supplementation reduced the protein and mRNA expression of both interleukin (IL)-6, and cyclooxygenase-2 (COX-2) (Figure 3A,B), and reduced the mRNA levels of IL-17, interferon (IFN-γ), and inducible nitric oxide synthase (iNOS) (Figure 3C) in the HFD-fed DSS-colitis mice. Altogether, these data confirmed the beneficial effect of GP via reducing inflammation and oxidative stress in DSS-colitis.

### 3.4. GP Supplementation Enhances MUC-2 Secretion and Goblet Cell Differentiation in HFD-Fed DSS-Colitis Mice

**Figure 4:**
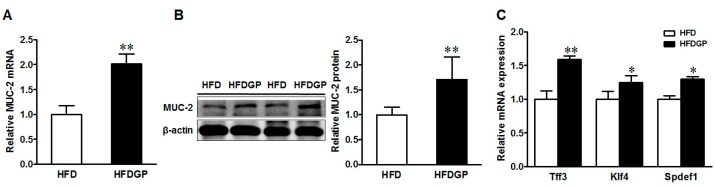
Mucin-2 and goblet cell differentiation markers in the colon of HFD or HFDGP fed DSS-colitis mice. (**A**) mRNA expression of MUC-2; (**B**) representative immunoblotting bands and statistical data of mucin 2; (**C**) mRNA expression of goblet cell differentiation markers, Tff3, Klf4, and Spdef1. Means ± SEM, *n* = 7, * *p* ≤ 0.05, ** *p* ≤ 0.01.

Mucin 2 is the major mucin produced by goblet cells and provides an additional protective layer to the gut epithelium. Both the mRNA and protein levels of mucin 2 were enhanced in the GP-supplemented HFD-fed DSS-treated mice (Figure 4A,B). In agreement, the gene expression of goblet cell differentiation markers including Trefoil factor 3 (Tff3), Krüppel-like factor 4 (Klf4), and SAM pointed domain ETS factor 1 (Spdef1) were higher in the GP-supplemented HFD-fed DSS-induced mice (Figure 4C). 

### 3.5. GP Supplementation Suppresses the Expression of Activating Transcription Factor-6 (ATF-6) and ER-Stress Markers in HFD-Fed DSS-Colitis Mice

**Figure 5:**
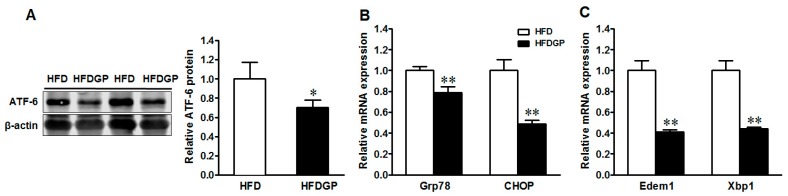
Endoplasmic reticulum (ER)-stress signaling in the colon of HFD or HFDGP fed DSS-colitis mice. (**A**) ATF-6 protein content; (**B**) mRNA expression of Grp78 and CHOP; (**C**) mRNA expression of Edem1 and Xbp1. Means ± SEM, *n* = 7, * *p* ≤ 0.05, ** *p* ≤ 0.01.

IBD is associated with ER stress and mucin 2 misfolding [24,25]. As part of the unfolded protein response (UPR), ATF-6 triggers the transcription of genes encoding the chaperone protein glucose-regulated protein 78 (Grp78), the CCAAT-enhancer-binding protein homologous protein (CHOP), the ER degradation-enhancing α-mannosidase-like 1 protein (Edem1), and the X-box binding protein 1 (Xbp1) [26,27]. Consistently, GP supplementation reduced the protein expression of ATF-6 (Figure 5A) and mRNA expression of its downstream target genes Grp78, CHOP (Figure 5B), Edem1, and Xbp1 in the HFD-fed DSS-colitis mice (Figure 5C), showing the suppression of ER stress. 

## 4. Discussion

Obesity is the root cause of many chronic diseases including diabetes, hypertension, and cardiovascular disease. Consumption of the HFD is associated with intestinal inflammation and increased permeability to the microbial end-products in mice [2,28]. The HFD enhances the severity of colitis in experimental colitis mice models [7,29,30], and promotes colon cancer initiation [31]. Further, inclusion of red meat in the Westernized HFD aggravated DSS-colitis in mice [4]. Peas are a valuable source of plant proteins, fiber, and polyphenolics [32], and its extract reduced inflammation in mice with DSS-induced colitis [17]. Our study shows that supplementation of GP accelerated the recovery from colitis symptoms in the HFD-fed mice as evident from decreased body weight loss and a lower fecal blood score during the recovery stage. In support of our findings, supplementation of dietary white and dark kidney beans as well as cranberry beans reduced colitis severity by reducing body weight loss, fecal blood score, and resultant DAI score in DSS-induced colitis mice [15,16]. Similarly, dietary supplementation of soybeans Bowman–Birk inhibitor concentrate reduced the severity of DSS-colitis by suppressing inflammation in the colon and improving the recovery following DSS-induction [14]. 

DSS causes mucosal and tissue damage in the mouse gut similar to the patterns of inflammatory responses observed in human UC [33,34]. The activation and infiltration of inflammatory cells, including neutrophils and monocytes, is one of the common features in colitis, which is a complex process driven by cytokines, chemokines, and cell adhesion molecules [35]. Cytokines mediate neutrophil infiltration into the intestinal wall and MCP-1, highly expressed in colonic mucosa in IBD [36], enhances the migration of neutrophils during chronic inflammation [23]. In DSS-induced colitis, Westernized HFD-feeding enhanced neutrophil infiltration as indicated by enhanced myeloperoxidase activity [4], and pea seed albumin extracts reduced inflammatory cell infiltration into the colon [17]. In agreement, the current study found that DSS-induction enhanced both the neutrophil infiltration and MCP-1 expression in the colon of HFD-fed mice, which were mitigated by GP supplementation. Inline, dietary white and dark kidney beans, as well as cranberry bean supplements, reduced the mRNA expression of MCP-1 in the colon of DSS-colitis mice [15,16]. 

Infiltrated neutrophils produce proinflammatory cytokines including IL-6, IL-17, and IFN-γ, and enhance the expression of oxidative stress enzyme, iNOS, further aggravating colitis [28,33]. IFN-γ plays an important role in the development of DSS-colitis, likely by activating and directing the leucocytes to the intestinal tissue [37]. Similarly, IL-17 stimulates epithelial cells to secrete IL-6 and helps CD34+ hematopoietic progenitors mature into neutrophils [38]. In the current study, elevated levels of IL-6, IL-17, IFN-γ, iNOS, and COX-2 caused by DSS-induction were ameliorated by GP supplementation in the colon of HFD-fed mice. Consistently, dietary white and dark kidney beans reduced the mRNA expression of IL-6 [15], and cranberry bean supplementation reduced the IL-6 protein in the colon along with reduced serum IL-6, IL-17, and IFN-γ in DSS-induced colitis [16]. The down-regulation of inflammatory cascades and oxidative stress by GP supplementation can be partially explained by the low neutrophil infiltration into the colon of HFD-fed DSS-colitis mice. 

The lubricating layer of mucus that shields the epithelium from the gut luminal content predominantly consists of mucin 2 produced by goblet cells. Mucin 2 goes through heavy extensive translational modifications in the ER and Golgi complex, making it susceptible to misfolding, and thus activating the UPR signaling [39]. Disturbance in the UPR and ER stress in intestinal epithelial cells induces chronic inflammation in IBD [24,25]. Missense mutations of the MUC-2 gene in *Winnie* and *Eeyore* mice increased ER-stress-related mucin depletion, resulting in colitis [8]. Recently, Gulhane and colleagues found that the HFD induced the expression of oxidative stress marker iNOS, and ER-stress markers including UPR signaling molecules Xbp1, ER chaperone Grp78, and ERAD chaperone Edem1 in the colon of *Winnie* mice [7]. On the other hand, dietary chickpea supplementation increased colon mucus content, mRNA expression of MUC-2, and differentiation marker Klf4 with enhanced gut barrier integrity and reduced inflammation in healthy unchallenged mice [13]. In DSS-induced colitis, dietary white and dark kidney beans, as well as dietary cranberry bean supplementation, enhanced the mRNA expression of MUC-2 and Tff3, and mitigated the severity of colitis and associated inflammation [15,16]. Consistent with these observations as well as improved colitis symptoms, GP supplementation improved both protein and gene expression of MUC-2 in HFD-fed DSS-induced mice, associated with the enhanced expression of goblet cell differentiation markers in the colon. Further, ATF-6 and its downstream ER-stress markers Grp78, CHOP, Edem1, and Xbp1 [24,25] were reduced in HFD-fed mice by GP supplementation. 

Legumes such as chickpeas, kidney beans, and cranberry beans contain dietary fiber, resistant starches, protein, and polyphenolics with reported beneficial effect on intestinal health [13,15,16]. The protein extract of soybeans and peas contains the active Bowman–Birk inhibitor that possesses anti-inflammatory activity and can reduce the severity of DSS-colitis in mice [14,17]. Using the whole food approach, we were not able to conclude which bioactive component in GP was responsible for protection against DSS-induced damages. Based on the previous investigations, the protective effect of GP can be attributed to the active Bowman–Birk inhibitor present in pea protein [14,17] and/or dietary fiber [12]. Dietary fiber in chickpea modulated the gut microbiota and enhanced SCFA production, correlating with improved gut epithelial barrier function [13]. These results suggested that GP might modulate gut microbiota to exert its protective effects on DSS-induced colitis.

## 5. Conclusions

GP supplementation reduces the severity of DSS-induced colitis in mice challenged with the HFD by reducing inflammation, mucosal loss, and the ER-stress signaling. GP possesses anti-inflammatory properties in DSS-induced colitis in mice fed a HFD, and can be used as a potential dietary management to reduce risk of IBD development.

## Supplementary Materials

The Supplementary Material are available online at www.mdpi.com/2072-6643/9/5/509/s1, Figure S1: An overview of experimental design, Figure S2: Feed intake and body weight of HFD and HFDGF fed mice before DSS-induction, Table S1: Composition of the experimental diets used in the study, Table S2: Primer sequences for quantitative reverse transcription PCR.
