# Supplementary material for: Dietary Green Pea Protects against DSS-Induced Colitis in Mice Challenged with High-Fat Diet
Source: Nutrients. 2017 May 18;9(5):509. doi: 10.3390/nu9050509 (PMC5452239; doi:10.3390/nu9050509)
Supplement: Supplementary file 1 [file nutrients-09-00509-s001.zip › nutrients-189464 suppl.pdf]

Supplementary Materials: Dietary green pea protects against DSS-induced colitis in mice challenged with high-fat diet

Shima Bibi, Luís Fernando de Sousa Moraes, Noelle Lebow, and Mei-Jun Zhu

Figure S1. An overview of experimental design. Six-week-old female mice were either fed HFD (45% energy from fat) or HFD supplemented with 10% GP (HFDGP) until necropsy. After the 7-week of dietary treatment, mice (13-weeks-old) in both groups were subjected to 2.5% DSS water for 7-days followed by a 7-days of recovery using normal water.

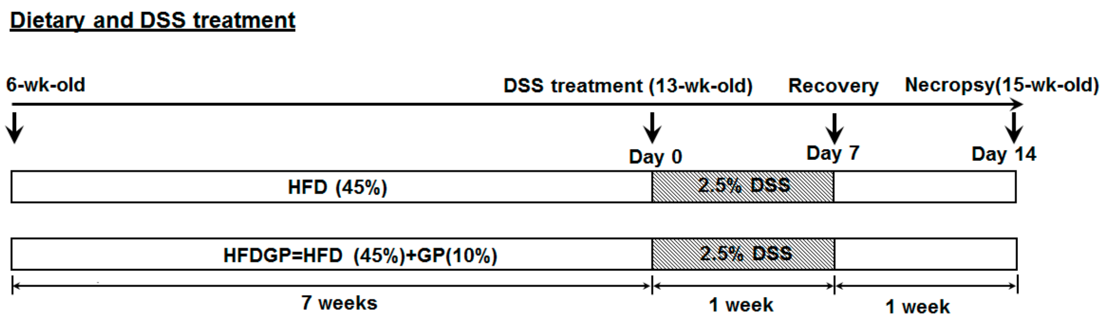

Figure S2. Feed intake (A), and body weight (B) of HFD (□) or HFDGP (■) fed mice before DSS-induction. Means ± SEM, n = 7.

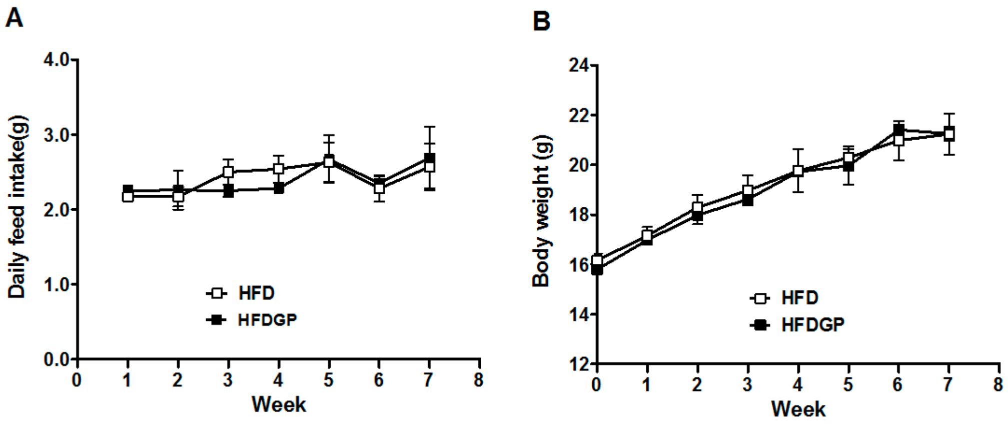

Table S1 Composition of the experimental diets[1]

Diet	45% HFD (D12451)		HFDGP[3] (D15080605)	
	**gm%**	**Kcal%**	**gm%**	**Kcal%**
Protein	24	20	24	20
Carbohydrates	41	35	41	35
Fat	24	45	24	45
Total		100		100
Kcal/gm	4.7		4.7	
**Ingredient**	**gm**	**kcal**	**gm**	**kcal**
Casein, 30 Mesh	200	800	179.58	718
L-Cystine	3	12	3	12
**Green Pea Powder (Protein)**[2]	0	0	**20.42**	82
Corn Starch	72.8	291	7.42	30
Maltodexrin 10	100	400	100	400
Sucrose	172.8	691	172.8	691
**Green Pea Powder (Carbohydrate)**[2]	0	0	**65.38**	262
Cellulose, BW200	50	0	50	0
Soybean Oil	25	225	25	225
Lard	177.5	1598	177.5	1598
Mineral Mix S10026	10	0	10	0
DiCalciun Phosphate	13	0	13	0
Calcium Carbonate	5.5	0	5.5	0
Potassium Citrate, 1 H_2O	16.5	0	16.5	0
Vitamin Mix V10001	10	40	10	40
Choline Bitartrate	2	0	2	0
FD&C red dye 40	0.05	0	0	0
FD&C blue dye 1	0	0	0.005	0
Total	858.15	4057	585.105	4057

[1]Diets were purchased from Research Diets Inc. (New Brunswick, NJ, USA) and information of diet composition was provided by the company.

[2]Grean Pea Powder contains 23.8% protein and 76.2% carbohydrates, of 25% dietary fiber. which were adjusted accordingly.

[3]Per USDA National Nutrient database, the mature split green pea contains 25.5% dietary fiber.

Table S2. Primer sequences for quantitative reverse transcription PCR

Gene Name	Accession No.	Product Size	Direction	Sequence (5'-3')	Source
CHOP	NM_007837.4	97bp	Forward	TCTTGAGCCTAACACGTCGAT	This study
			Reverse	TGGAACACTCTCTCCTCAGGT	
COX-2	NM_011198.3	169bp	Forward	AGCCAGGCAGCAAATCCTT	[1]
			Reverse	GGGTGGGCTTCAGCAGTAAT	
Edem1	NM_138677.2	114bp	Forward	GGCATGTTCGTCTTCGGCTA	This study
			Reverse	CAGATTGGAAGGGTCTCCGC	
Grp78	NM_022310.3	272bp	Forward	TTCGTGTCTCCTCCTGACCC	This study
			Reverse	GAACACACCGACGCAGGAA	
IFN-γ	NM_008337.3	93 bp	Forward	AGGTCCAGCGCCAAGCATTCAA	[2]
			Reverse	AGCAGCGACTCCTTTTCCGCTT	
IL-6	M20572	141bp	Forward	GAGGATACCACTCCCAACAGACC	[3]
			Reverse	AAGTGCATCATCGTTGTTCATACA	
IL-17A	NM_010552.3	142bp	Forward	GCTCCAGAAGGCCCTCAGA	[3]
			Reverse	AGCTTTCCCTCCGCATTGA	
iNOS	U43428	95 bp	Forward	CAGCTGGGCTGTACAAACCTT	[3]
			Reverse	CATTGGAAGTGAAGCGTTTCG	
Klf4	NM_010637.3	75 bp	Forward	CAGGATTCCATCCCCATCCG	[4]
			Reverse	GAGAGGGGACTTGTGACTGC	
MCP-1	NM_011333.3	117bp	Forward	CACTCACCTGCTGCTACTCA	This study
			Reverse	GCTTGGTGACAAAAACTACAGC	
MUC-2	NM_023566.2	101bp	Forward	ATGCCCACCTCCTCAAAGAC	This study
			Reverse	GTAGTTTCCGTTGGAACAGTGAA	
Spdef1	NM_013891.4	280bp	Forward	CTTGTCATGGTGCCAGCAG	This study
			Reverse	TTGGGGCTGCTTCCGTTA	
Tff3	NM_011575.2	103bp	Forward	CTGTCACATCGGAGCAGTGT	This study
			Reverse	AATGTGCATTCTGTCTCCTGC	
Xbp1	NM_001271730.1	118bp	Forward	TACGGGAGAAAACTCACGGC	This study
			Reverse	CTTACTCCACTCCCCTTGGC	
18S rRNA	NR_003278.1	122bp	Forward	AAGACGGACCAGAGCGAAAG	This study
			Reverse	ATCGCCAGTCGGCATCGTTT	

References

1. Kang, Y.; Xue, Y.; Du, M.; Zhu, M.J. Preventive effects of goji berry on dextran-sulfate-sodium-induced colitis in mice. *J Nutr Biochem* **2016**, *40*, 70-76.
2. Wang, H.; Xue, Y.; Zhang, H.; Huang, Y.; Yang, G.; Du, M.; Zhu, M.J. Dietary grape seed extract ameliorates symptoms of inflammatory bowel disease in il10-deficient mice. *Mol Nutr Food Res* **2013**, *57*, 2253-2257.

3. Giulietti, A.; Overbergh, L.; Valckx, D.; Decallonne, B.; Bouillon, R.; Mathieu, C. An overview of real-time quantitative pcr: Applications to quantify cytokine gene expression. *Methods (San Diego, Calif.)* **2001**, *25*, 386-401.
4. Yang, G.; Xue, Y.; Zhang, H.; Du, M.; Zhu, M.J. Favourable effects of grape seed extract on intestinal epithelial differentiation and barrier function in il10-deficient mice. *Br J Nutr* **2015**, *114*, 15-23.
